# Fertility desire and intention of people living with HIV/AIDS in Tanzania: a call for restructuring care and treatment services

**DOI:** 10.1186/1471-2458-13-86

**Published:** 2013-01-30

**Authors:** Elia J Mmbaga, Germana H Leyna, Mangi J Ezekiel, Deodatus C Kakoko

**Affiliations:** 1 Department of Epidemiology and Biostatistics, Muhimbili University of Health and Allied Sciences, P.O.Box 65015, Dar Es Salaam, Tanzania; 2 Department of behavioural Sciences, Muhimbili University of Health and Allied Sciences, Dar Es Salaam, Tanzania

## Abstract

**Background:**

Scaling up of antiretroviral therapy (ART) is currently underway in sub-Saharan Africa including, Tanzania, increasing survival of people living with HIV/AIDS (PLWHA). Programmes pay little attention to PLWHA’s reproductive health needs. Information on fertility desire and intention would assist in the integration of sexual and reproductive health in routine care and treatment clinics.

**Methods:**

A cross-sectional study of all PLWHA aged 15–49 residing in Kahe ward in rural Kilimanjaro Tanzania was conducted. Participants were recruited from the community and a local counselling centre located in the ward. Data on socio-demographic, medical and reproductive characteristics were collected through face-to-face interviews. Data were entered and analysed using STATA statistical software.

**Results:**

A total of 410 PLWHA with a mean age of 34.2 and constituting 264 (64.4%) females participated. Fifty-one per cent reported to be married/cohabiting, 73.9% lived with their partners and 60.5% were sexually active. The rate of unprotected sex was 69.0% with 12.5% of women reporting to be pregnant at the time of the survey. Further biological children were desired by 37.1% of the participants and lifetime fertility intention was 2.4 children. Increased fertility desire was associated with living and having sex with a partner, HIV disclosure, good perceived health status and CD4 count ≥200 cells for both sexes. Reduced desire was associated with havingmore than 2 children among females, divorce or separation, and having a child with the current partner among both males and females.

**Conclusion:**

Fertility desire and intention of PLWHA was substantially high though lower than that of the general population in Tanzania. Practice of unprotected sexual intercourse with higher pregnancy rate was observed. Fertility desire was determined by individual perceived health and socio-family related factors. With increasing ART coverage and subsequent improved quality of life of PLWHA, these findings underscore the importance of integrating reproductive health services in the routine care and treatment of HIV/AIDS worldwide. The results also highlight a group of PLWHA with potentially high desire for children who need to be targeted during care.

## Background

An estimated total of 33 million people are currently living with HIV/AIDS worldwide. Sub-Saharan Africa with only 10% of the population of the world is home to 60% of people living with HIV/AIDS (PLWHA) [1]. Tanzania is among the countries severely affected by the epidemic with 5.7% of the 40 million population infected with the HIV virus[2]. Antiretroviral therapy (ART) has improved health status and life expectancy of PLWHA making them enjoy life similar to uninfected individuals [3-6]. Some studies have even reported that some antiretroviral drugs may increase sexual activity among women increasing their likelihood of pregnancies [7]. Scaling up of ART treatment is currently underway in sub-Saharan Africa increasing survival of PLWHA with little attention on their reproductive health needs [1]. Unprotected sex has been discouraged among PLWHA due to risk of transmission or acquisition of new viral strains and vertical transmission. Policies and stigma have discouraged reproductive intentions of PLWHA. However, studies suggest that PLWHA desire and continue to have children equally to those without HIV infection [8,9]. A limited number of studies mostly in developed countries involving selected groups of people such as women in ART centres and/or urban settings where socio-cultural pressures are less have speculated that most of pregnancies of PLWHA could be intentional [9-12]. In rural areas, cultural values are attached to fertility and a significant social status is assigned to people with children [13]. In this era of HIV infection, the level of desire and intention to have children in the rural general population is not well understood. Additionally, there are a few insights in to factors affecting desire and intentions to have children among PLWHA in Africa. Lack of information has slowed down efforts to integrate reproductive health services in the routine HIV/AIDS care and treatment.

The study aimed at estimating the size of fertility desire and intention, and to identify factors associated with fertility desire among PLWHA. The findings can be used to promote efforts for provision of reproductive health services in HIV care and treatments clinics in Tanzania.

## Methods

### Design and population

A cross-sectional study of PLWHA residing in Kahe ward, rural Kilimanjaro region of Tanzania was conducted between January and February of 2010. All PLWHA aged 15–49 identified from our previous incidence and sero-prevalence study [14,15] in the area and all others who live and receive services at local non-governmental HIV counselling group (KIWAKKUKI) in the ward were involved in this study. The group offer the same albeit better quality services to PLWHA as those provided by public sector. These services include individual counselling on drug adherence and nutrition due to few and manageable number of patients as compared to public health facilities.

### Study area

The study took place in Kahe ward Moshi Rural District in the Kilimanjaro region of Tanzania. PLWHA living in all 11 villages of the Kahe ward were involved in this study. The study area is located about 30 km southeast of Moshi town. This rural population was chosen because it is among rural areas in Tanzania with evidence of higher incidence and prevalence of HIV infection. Moreover, our previous works in the area identified and built a good relationship with PLWHA and ward authority which proved helpful in the tracing and identification of PLWHA.

### Subject recruitment and interviews

All PLWHA identified from our earlier surveys who received counselling and nutrition support from KIWAKKUKI group and all others diagnosed before or after our survey who received services in the group or living in the study area were enrolled in this study. These individuals were receiving continued counselling, nutrition support, ART and follow-up in the group. We used a list of all members of the group and assistance from the community leaders and Community Health Outreach staff to trace other PLWHA who were not registered but living in the study area.

In collaboration with staff of the counselling group, the researchers made appointments with all PLWHA attending the centre and an interview scheduled during their normal clinic days. A structured questionnaire was administered to all consenting participants in a face –to-face interview by trained interviewers. Data on social demographic characteristics, reproductive history, fertility desire (expecting to have children in the future), and fertility intention (number of children intended in the future), medical condition (CD4 count, ART status) and perceived health and HIV transmission knowledge were collected. All participants who did not turn up on their scheduled clinic were visited at home through their community health outreach staff. We managed to recruit all 384 participants listed in the group and further identified 26 other PLWHA in the community who were not registered in the group. These had definitive HIV test results and were receiving care outside the ward.

### Data analysis

During the data collection process, completed questionnaires were checked daily for completeness and consistency. Data were entered in to a computer using statistical software for Social scientists (SPSS version 15). All analyses were done using STATA version 11 statistical software. An HIV transmission knowledge scale was constructed from 8 questions asking various aspect of transmission including both horizontal and vertical transmission. Each correct answer was awarded a score of one and incorrect answers given a score of zero. The scale score ranged from 0–8, mean score 6.2 with a Chronbach’s alpha of 0.814. The scale was dichotomised along the mean with those scoring from the mean and above considered knowledgeable and the rest not knowledgeable. Frequencies were generated for all categorical variables and differences between proportions were examined using the Chi-square test for differences in proportions. Continuous variables were summarized using means and standard deviation and differences between means examined using *t*-test. Desire for children was set as an outcome variable and both bivariate and multivariate analysis of determinants of fertility desire explored. All independent variables achieving a p-value below 0.2 were included in the final logistic regression model. Backward stepwise logistic regression method was used with model fit assessed based on the log-likelihood ratio test. Adjusted odds ratio and their corresponding 95% confidence intervals were calculated in the final multivariate models and are reported. All the analysis were two tailed and type 1 error rate was set at 5% level.

### Ethical consideration

The ethical committee of the Muhimbili University of Health and Allied Sciences approved the final study protocol. Permission was also granted from the District, ward and village authorities. All participants gave their written informed consent before participation. All the interviews were conducted in a secure and private place with no names recorded.

## Results

A total of 410 PLWHA aged between 18 and 49 were identified and recruited in the study. The overall mean age was 34.2 (standard deviation (SD), 7.6), with males significantly older than females (Mean 36.7 ± 7.6 for males and 32.7 ± 7.2 for females, p < 0.001). Females constituted 64.4% (264) of all study participants.

### Socio-demographic characteristics of the participants

The distribution of participant’s education, occupation and marital statuses did not differ by sex. Half of the participants (51.5%) reported to have been married or cohabiting with a partner. The reported mean age at marriage/cohabiting was significantly higher among males (24.3 ± 4.5) than that females (20.7 ± 3.6), p < 0.001) with majority of males marrying after 18 years (p = 0.003). Of the participants who reported to be married or cohabiting at the time of the survey, nearly three quarters (73.9%) reported to be living with their partners, and this did not differ by sex. Current reported number of children did not differ between male and female participants. A large majority of males (80.6%0 than female (67.4%) participants reported having children with their current partners (Table 1).

**Table 1:** Distribution of socio-demographic characteristics of the study participants by sex

**Variable**	**Category**	**Male**	**Female**	
		**n(%)**	**n(%)**	**p-value**
Age group	15-24	7(4.8)	38(14.4)	<0.001
	25-34	47(32.2)	113(42.8)	
	35-44	59(40.4)	99(37.5)	
	≥45	33(22.6)	14(5.3)	
Education	No education	14(9.6)	21(8.0)	0.583
	Primary	93(63.7)	184(69.7)	
	Secondary	22(15.1)	37(14.0)	
	Post-secondary	17(11.6)	22(8.3)	
Marital status	Never married	16(11.0)	36(1.6)	0.265
	Married/Cohabiting	83(56.8)	128(48.5)	
	Separa/Divorced	47(32.2)	100(37.9)	
Occupation	Farmer	74(50.7)	131(49.6)	0.955
	Employed	20(13.7)	35(13.3)	
	Business	52(5.6)	98(37.1)	
Age at first marriage/cohabiting‡	<18 years	4(3.0)	28(12.2)	0.003
	≥18 years	127(97.0)	201(87.8)	
Live with partner†	Yes	58(69.9)	98(76.6)	0.280
	No	25(30.1)	30(23.4)	
No of children	0	36(24.7)	70(26.5)	0.242
	1	26(17.8)	63(23.9)	
	≥2	84(57.5)	131(49.6)	
Having sex with partner†	Yes	90(61.6)	158(59.8)	0.722
	No	56(38.4)	106(40.2)	
Children with current partner†	Yes	54(80.6)	58(67.4)	0.068
	No	13(19.4)	28(32.6)	

‡: for those who reported to have been married/cohabiting, †; for only those reporting to have a sexual partner, *ART *Antiretroviral Therapy, *SD *Standard deviation.

### Medical related characteristics of the study population

The level of HIV disclosure was found to be high in this population with 86.3% (354) of participants reporting to have disclosed their HIV status either to their spouses, family members or friends (Table 2). Participants disclosed more their HIV sero-statuses to their family members and to their partners. The majority of males (80.7%) reported their current sexual partners to have tested for HIV as compared to female participants (59.1%) (p = 0.002). About half of the participants, similar for males and females, perceived their health status to be good. With regards to HIV transmission knowledge, a substantial proportion of the participants (68.0%) were knowledgeable about HIV transmission both horizontal and vertical and this did not differ by sex.

**Table 2:** Distribution of medical related characteristics of study participants by sex

**Variable**	**Category**	**Male**	**Female**	
		**n(%)**	**n(%)**	**p-value**
Disclosed HIV status	Partner	29(19.9)	53(20.1)	0.303
	Family member	84(57.5)	142(53.8)	
	Friends	19(13.0)	27(10.2)	
	None	14(9.6)	42(15.9)	
Partner tested†	Yes	71(80.7)	94(59.1)	0.002
	No	13(14.8)	53(33.3)	
	Don’t know	4(4.5)	12(7.6)	
Know partner HIV status†	Yes	49(69.0)	63(67.0)	0.221
	No	22(31.0)	31(33.0)	
Perceived health status	Good	75(51.4)	147(55.7)	0.401
	Poor	71(48.6)	117(44.3)	
HIV knowledge	Knowledgeable	108(74.0)	188(71.2)	0.550
	Not knowledgeable	38(26.0)	76(28.8)	
On ART	Yes	98(67.1)	162(61.4)	0.246
	No	48(32.9)	102(38.6)	
CD4 count	<200	55(37.7)	90(34.1)	0.468
	≥200	91(62.3)	174(65.9)	
Mean CD4 count	Mean(SD)	275(186)	320(222)	0.978
Having sex with partner†	Yes	90(61.6)	158(59.8)	0.722
	No	56(38.4)	106(40.2)	
Desire for more children	Yes	58(39.7)	94(35.6)	0.408
	No	88(60.3)	170(64.4)	
No. desired children(Intention)	Mean(SD)	2.4(1.5)	2.5(1.6)	0.672
Condom use during last sex†	Yes	20(22.5)	52(32.7)	0.089
	No	69(77.5)	107(67.3)	
Currently pregnant	Yes		34(12.9)	
	No		230(87.1)	

‡: for those who reported to have been married/cohabiting, †; for only those reporting to have a sexual partner, *ART *Antiretroviral Therapy, *SD *Standard deviation.

A total of 260 (63.3%) participants reported to be on ART at the time of the survey and the mean duration of ART use was 4.9 years. The mean CD4 count of the study participants was 304 cells and 264 (64.6%) participants reported to have CD4 count ≥200 cells. Perceived health status, ART use status and CD4 cell did not differ by sex (Table 2).

### Fertility desire and intention

**Figure 1:**
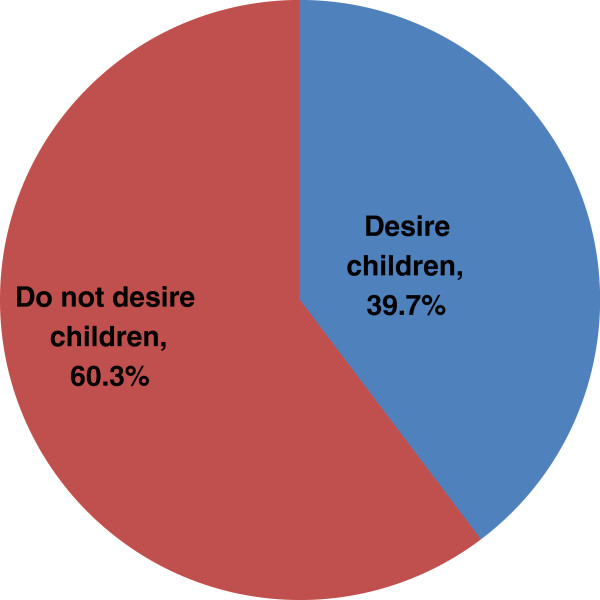
Distribution of Fertility desire among PLWHA in rural Kilimanjaro, Tanzania.

Of the participants interviewed, 60.5% (248) reported to engage in sexual activities Desire for a child or additional child was expressed by 37.1% (152) of the participants (39.7% of males and 35.6% of females) (p = 0.408) (Figure 1). The mean lifetime desired number of children was 2.4 ± 1.4 and this did not differ between males and females (2.4 ± 1.5 versus 2.5 ± 1.6, p = 0.672). At the time of the survey, 12.4% of women reported to be pregnant (Table 2).

### Factors associated with fertility desire

Table 3 presents the association between different socio-demographic, medical and reproductive factors and desire for children in the study population. A significantly large proportion of study participants who reported to never married, those living with partners, those reported to have sex with their partners, those with no or 1 child, those who did not have a child with the current partner, those who disclosed their HIV status, those who perceived to have good health status and those who had CD4 count above 200 cells were more likely to desire for children (Table 3).

**Table 3:** Association between fertility desire and socio-demographic and medical characteristics of study participants

**Variable**	**Category**	**Desire a child**	**Do not desire a child**	**OR**	**P value**
		**n (%)**	**n (%)**		
Age group	15-24	18(40.0)	27(60.0)	1	0.069
	25-34	70(43.8)	90(56.2)	1.12	
	35-44	52(32.9)	106(67.1)	0.83	
	≥45	12(25.5)	35(74.5)	0.65	
Sex	Male	58(39.7)	88(60.3)	1	0.408
	Female	94(35.6)	170(64.4)	0.73	
Education	No education	11(31.4)	24(68.6)	1	0.091
	Primary	112(40.4)	165(59.6)	1.68	
	Secondary	21(35.6)	38(64.4)	1.22	
	Post-secondary	8(20.5)	31(79.5)	0.64	
Occupation	Farmer	69(33.7)	136(66.3)	1	0.136
	Employed	18(32.7)	37(67.3)	1.00	
	Business	65(43.3)	85(56.7)	1.51	
Marital status	Never married	25(48.1)	27(51.9)	1	0.050
	Married/Cohabiting	82(38.9)	129(61.1)	0.64	
	Separa/Divorced	45(30.6)	102(69.4)	0.30	
Age at first marriage/cohabiting‡	<18 years	16(50.0)	16(50.0)	1	0.080
	≥18 years	113(34.5)	215(65.5)	0.55	
Live with partner	Yes	101(64.7)	28(50.9)	1	<0.001
	No	55(35.4)	27(49.1)	1.77	
No of children	0	46(43.4)	60(56.6)	1	0.048
	1	37(41.6)	52(58.4)	0.87	
	≥2	69(32.1)	146(67.9)	0.51	
Having sex with partner†	Yes	105(42.)	143(57.7)	1	0.006
	No	47(29.0)	115(71.0)	1.80	
Condom use during last sex†	Yes	77(43.8)	99(56.2)	1	0.482
	No	28(38.9)	44(61.1)	1.22	
Children with current partner†	Yes	24(44.0)	31(56.0)	0.51	<0.001
	No	94(60.0)	62(40.0)	1	
Disclosed HIV status	Yes	141(39.8)	213(60.2)	1	0.004
	No	11(19.6)	45(80.4)	4.13	
Partner tested†	Yes	71(43.0)	94(57.0)	1	0.815
	No	34(41.5)	48(58.5)	0.89	
Know partner status†	Yes	47(42.0)	65(58.0)	1	0.688
	No	24(45.3)	29(54.7)	1.11	
Perceived health status	Good	97(43.7)	125(56.3)	1	0.003
	Poor	55(29.3)	133(70.7)	2.01	
HIV knowledge	Knowledgeable	114(38.5)	182(61.5)	1	0.331
	Not knowledgeable	38(33.3)	76(66.7)	0.78	
On ART	Yes	104(40.0)	156(60.0)	1	0.106
	No	48(32.0)	102(68.0)	0.64	
CD4 count	<200	40(28.3)	104(71.7)	1	0.006
	≥200	111(41.9)	54(58.1)	1.96	

‡: for those who reported to have been married/cohabiting, †; for only those reporting to have a sexual partner, ART; Antiretroviral Therapy, OR; unadjusted odds ratio.

Multivariate analyses revealed that males and females who were separated or divorced were 50% and 70% less likely to desire children in the future, respectively (Table 4). Males who were living with their partners were two and a half times more likely to desire children than those not living with partners. Similar observation was reported for females (AOR, 1.9, 95%CI: 1.0; 4.7). Having more than two children was associated with reduced likelihood of desiring more children among females (AOR, 0.4, 95%CI: 0.2; 0.8) but not among men. Having sex with the current parner was associated with increased desire for children in the future for both male (AOR, 4.3, 95%CI: 1.2; 8.7) and female (AOR, 5.9. 95%CI: 1.7; 20.3) while having a child with current partner was associated with a 50% and 60% lower likelihood of desiring for a child in the future for females and males, respectively. Additionally, participants who disclosed their HIV statuses, having good perceived health status and CD4 count ≥200 cells were more likely to desire for children (Table 4).

**Table 4:** Independent determinants of fertility desire among male and female living with HIV/AIDS in rural Kilimanjaro, Tanzania

**Variable**	**Category**	AOR(95%CI)*	**P value**	AOR(95%CI)*	**p-value**
		**Male**		**Female**	
Age group	15-24	1		1	
	25-34	1.1(0.7;2.3)	0.765	0.9(0.5;2.2)	0.979
	35-44	0.7(0.3;1.2)	0.317	0.3 (0.2;1.4)	0.071
	≥45	0.5(0.2;1.1)	0.092	0.4(0.1;1.2)	0.061
Education	No education	1		1	
	Primary	1.2(0.3;4.2)	0.697	1.0(0.3;3.0)	0.914
	Secondary	0.9(0.2;4.2)	0.989	0.7(0.2;2.6)	0.685
	Post-secondary	0.3(0.1;1.9)	0.252	0.3(0.1;1.5)	0.164
Marital status	Never married	1		1	
	Married/Cohabiting	0.7(0.2;2.5)	0.709	0.6(0.2;1.3)	0.203
	Separa/Divorced	0.5(0.2-0.9)	0.046	0.3(0.2;0.7)	0.005
Occupation	Farmer	1		1	
	Employed	0.9(0.2;4.1)	0.956	0.7(0.1;3.2)	0.728
	Business	1.6(0.5;4.9)	0.375	2.2(0.9;5.0)	0.053
Age at first marriage/cohabiting‡	<18 years	1		1	
	≥18 years	1.2(0.6;5.2)	0.434	0.2(0.1;1.6)	0.081
Live with partner†	No			1	
	Yes	2.5(1.2;3.5)	0.007	1.9(1.0;4.7)	0.023
No of children	0	1		1	
	1	0.8(0.2;2.4)	0.763	0.6(0.2;1.3)	0.208
	≥2	0.6(0.1;1.5)	0.344	0.4(0.2;0.8)	0.009
Having sex with partner†	No	1		1	
	Yes	4.3(1.2;8.7)	0.004	5.9(1.7;20.3)	0.005
Children with current partner†	No	1		1	
	Yes	0.5(0.1;0.8)	0.042	0.4(0.2;0.9)	0.034
Disclosed HIV status	No	1		1	
	Yes	9.8(1.2;7.7)	0.030	4.2(1.5;6.9)	0.001
Perceived health status	Poor	1		1	
	Good	2.1(1.0;4.4)	0.044	1.7(1.0;3.0)	0.037
On ART	No	1		1	
	Yes	0.4(0.1;2.1)	0.295	0.9(0.3;2.3)	0.900
CD4 count	<200	1		1	
	≥200	1.7(1.1;4.3)	0.007	2.0(1.1;3.7)	0.015

‡: for those who reported to have been married/cohabiting, †; for only those reporting to have a sexual partner; *AOR Adjusted Odds Ratio-adjusted for other variables in the table, *CI *Confidence Interval, ART, Antiretroviral Therapy.

## Discussion

A total of 410 PLWHA with mean age of 34.2 were recruited and interviewed in this study. Half of the participants were in relationship with 73.9% of them living with their sexual partners. About 60% of the participants reported to engage in sexual act with their partners with only 29.0% reported condom use during last sexual act. In this study, 37% of the respondent desired a child or additional child and the fertility intention was 2.4 children. Positive predictors of fertility desire were living with partner, disclosure of HIV status, having sex, not having more than one child with the partner, perceived good health status and CD4 cell count ≥200 cells. Negative predictors of fertility desire were being divorced or separated (both sexes), and having ≥2 children (for females).

Over 80% of people living with HIV/AIDS are in their reproductive years and many continue to want children after learning of their positive status (whether to start a family or to have more children) [1,2]. It is therefore imperative to examine their reproductive health needs to be able to design programmes for safe reproduction while reduced HIV transmission.

In this study, the level of fertility desire was similar with what was reported in other studies elsewhere [16]. In contract, our estimate was relatively higher than the 29% reported in South Africa [17]. On the other hand, our estimate was lower than that of the general population in Tanzania which is estimated to be 60% [18]. This could partly be explained by previous efforts to discourage child bearing among PLWHA in most countries [19,20].

Our study population largely constituted of PLWHA who were enrolled in a special counselling group where they received counselling on safe sex, drug adherence and family planning services and this might have impacted safer reproductive health knowledge and practice among the participants. This could also explain the observed lower fertility desire and intention estimates as compared to the general population.

Lifetime fertility desire of 2.4 children in this population was substantially lower than that of the general population of 5.4 [18]. This could be explained by the fact that PLWHA have lower desire for children with reasons explained above as well as other medical and psychosocial reasons[21,22]. Moreover, there has been a general decline in fertility intention in the general population in Tanzania from 5.7 in 2005 to 5.4 in 2010 [18].

For public health purposes, a fertility desire of 37% amongst PLWHA is higher enough to warrant a special attention by promoting a “one-stop shopping” for both HIV/AIDS care and reproductive health services for PLWHA. This is corroborated by the unprotected nature of sexual activity reported in this population (69%) which indicates a potential for HIV transmission in the case of discordant couples. This is substantiated by the pregnancy rate of 12.5% in this population which is on the higher side as compared to that of the general population (9-10%)[18]. Although we did not directly establish what proportion of these pregnancies were intentional, the relatively higher desire for children indicate that majority of these pregnancies could be intentional. All pregnant women in Tanzania are offered voluntary counselling and testing and those testing positive are enrolled in the prevention of mother to child transmission programmes. However, no special reproductive health services are available in care and treatment clinic to offer appropriate services to PLWHA [22]. The programmes to be developed should address one stop access to contraception methods, counselling on reproductive related decision and safer conception, pregnancy, and delivery.

As expected, we found that younger participants and those who were never married or in marriage to have relatively higher desire for children in this population. This finding conforms to findings reported in Nigeria and South Africa [23,24]. Higher fertility desires among married individuals can be explained by the social expectation of marriage as reported elsewhere in Tanzania and beyond [18,23]. Moreover, younger unmarried individual who are still in their early reproductive age would be expected to desire children than older ones who are more likely to already have children. In the current shortage of human resource for health in the country, this study indicates that a focus on younger individuals would be beneficial. Health personnel could identify and offer special reproductive health services to these individuals as they attend care and treatment clinics.

Previous studies have shown that higher self-ratings of overall personal health status and physical functioning were associated with increased fertility desires [6,23]. These previous reports support the findings in this study where perceived health status was found to be associated with fertility desire. Having CD4 cells ≥200 cells is clinically associated with better health hence normal sexual activity and desire for family. Higher number of CD4 cells is a surrogate measure of a stronger immunity and better health status among PLWHA.

It has been observed in other studies that not having own children to be an important determinant of increased fertility desires [23,25-27]. This was also observed in this study where a tendency to desire children was higher for those participants who did not have children or those without children with current partner. Participants with more than two children, the intended number of children, had 40% lower likelihood to desire children as compared to those without children as reported in other previous studies [6,17,24].

Disclosure of HIV status was associated with desire for children in this population. HIV disclosure, open doors to support, access to counselling and reproductive health information and options to facilitate the ability to make informed decision on children bearing [24,28].

We found a non-significant association between ART use and fertility desire in this study. This may indicate that, regardless of ART status of the person, actual feeling, physical status of the person and socio-family related predictors such as number of children, having children with the partner and age are more important drivers of fertility desire[6,29]. As reported in other studies, [21] the actual use of ART may not have a greater impact on fertility desires but rather the optimism of longer life due to ART could impact more on the desire.

The interpretation of the findings of this study should consider the following potential limitations; Firstly, the cross sectional nature of this study may limit the causal and effect interpretation of the factors observed. Secondly, reported behaviours especially those related to sexual life may be affected by desirability bias. Lastly, the fact that our population had an opportunity for counselling in the KIWAKKUKI group with better services than those offered in the public sector, and this may have increased their reproductive risk assessments affecting their desire.

## Conclusion

A substantial proportion of PLWHA living in rural Tanzania continue to desire children albeit at lower rate than that of the general population. Practice of unprotected sexual intercourse with high pregnancy rate was observed. Fertility desire seems to be determined more by perceived health status and socio-family related factors. With increasing ART coverage that improves health status of PLWHA, more unprotected sex and pregnancies shall be expected. These findings provide more evidence for the need to increase efforts to integrate reproductive health services (contraception, counselling on decision to have a child and safer conception) in the routine HIV/AIDS care and treatment in sub Saharan countries and beyond. These strategies my not only improve reproductive health outcomes of PLWHA but also complement on going HIV prevention efforts.

## Competing interests

The authors declare that they have no competing interest.

## Authors’ contributions

EJM- designed the study, drafted the manuscript, collected the data, analyzed and reviewed the final manuscript. GHL-designed the study, assisted in the interpretation of the results and reviewed the final manuscript, MJE and DCK both participated study design, interpretation of the results and reviewed the final manuscript. All authors read and approved the final draft of the manuscript.

## Pre-publication history

The pre-publication history for this paper can be accessed here:

http://www.biomedcentral.com/1471-2458/13/86/prepub
